# Comparative sequencing analysis reveals high genomic concordance between matched primary and metastatic colorectal cancer lesions

**DOI:** 10.1186/s13059-014-0454-7

**Published:** 2014-08-28

**Authors:** A Rose Brannon, Efsevia Vakiani, Brooke E Sylvester, Sasinya N Scott, Gregory McDermott, Ronak H Shah, Krishan Kania, Agnes Viale, Dayna M Oschwald, Vladimir Vacic, Anne-Katrin Emde, Andrea Cercek, Rona Yaeger, Nancy E Kemeny, Leonard B Saltz, Jinru Shia, Michael I D’Angelica, Martin R Weiser, David B Solit, Michael F Berger

**Affiliations:** Department of Pathology, Memorial Sloan Kettering Cancer Center, New York, NY 10065 USA; Human Oncology and Pathogenesis Program, Memorial Sloan Kettering Cancer Center, New York, NY 10065 USA; Department of Medicine, Memorial Sloan Kettering Cancer Center, New York, NY 10065 USA; Department of Surgery, Memorial Sloan Kettering Cancer Center, New York, NY 10065 USA; Genomics Core Laboratory, Memorial Sloan Kettering Cancer Center, New York, NY 10065 USA; New York Genome Center, New York, NY 10065 USA; Weill Cornell Medical College, New York, NY 10065 USA

## Abstract

**Background:**

Colorectal cancer is the second leading cause of cancer death in the United States, with over 50,000 deaths estimated in 2014. Molecular profiling for somatic mutations that predict absence of response to anti-EGFR therapy has become standard practice in the treatment of metastatic colorectal cancer; however, the quantity and type of tissue available for testing is frequently limited. Further, the degree to which the primary tumor is a faithful representation of metastatic disease has been questioned. As next-generation sequencing technology becomes more widely available for clinical use and additional molecularly targeted agents are considered as treatment options in colorectal cancer, it is important to characterize the extent of tumor heterogeneity between primary and metastatic tumors.

**Results:**

We performed deep coverage, targeted next-generation sequencing of 230 key cancer-associated genes for 69 matched primary and metastatic tumors and normal tissue. Mutation profiles were 100% concordant for *KRAS*, *NRAS*, and *BRAF*, and were highly concordant for recurrent alterations in colorectal cancer. Additionally, whole genome sequencing of four patient trios did not reveal any additional site-specific targetable alterations.

**Conclusions:**

Colorectal cancer primary tumors and metastases exhibit high genomic concordance. As current clinical practices in colorectal cancer revolve around *KRAS*, *NRAS*, and *BRAF* mutation status, diagnostic sequencing of either primary or metastatic tissue as available is acceptable for most patients. Additionally, consistency between targeted sequencing and whole genome sequencing results suggests that targeted sequencing may be a suitable strategy for clinical diagnostic applications.

**Electronic supplementary material:**

The online version of this article (doi:10.1186/s13059-014-0454-7) contains supplementary material, which is available to authorized users.

## Background

Precision oncology relies on the accurate characterization of targetable oncogenic mutations present at the time of metastatic disease. However, it is often challenging to obtain biopsies of metastatic tumors, and it is still preferable to use the least invasive screening methods possible. Emerging evidence of both inter- and intra-tumor genetic heterogeneity in several solid tumor types raises concerns that molecular profiling of primary tumors may not be representative of metastatic disease [1-3]. In colorectal cancer (CRC), comparative lesion sequencing of a small number of cases found a high degree of concordance between primary tumors and metastases [4]. In contrast, a recent study of 21 patients using next generation sequencing reported a high degree of mutational discordance between primary and metastatic samples [5].

We previously showed that when analysis was performed on the invasive compartment of primary tumors, KRAS, NRAS, BRAF, and TP53 mutations were highly concordant between primary and metastatic tumors [6]. This study provided a preliminary indication that the use of archived primary tumor for molecular profiling may be suitable for clinical decision making in metastatic CRC. However, this conclusion was based on the analysis of only a small number of genes by mass-spectrometry based genotyping and Sanger sequencing.

To determine the extent of additional, clinically relevant genetic heterogeneity, we extended this analysis by performing high coverage, next generation sequencing analysis of 230 cancer-associated genes. Specifically, we performed targeted sequencing on primary, metastatic, and normal tissue from 69 colorectal cancer patients. We found that there was a high degree of concordance with regard to early occurring and recurrent mutations. KRAS, NRAS, and BRAF mutations were always identical in both the primary and metastatic tumors. Whole genome sequencing of two concordant and two discordant patient sets upheld the targeted sequencing results and revealed few additional recurrent mutations. In sum, these data suggest that for current clinical practices, either primary or metastatic tissue can be selected for testing, and targeted sequencing of key cancer genes is a suitable strategy for identifying clinically actionable alterations.

## Results

### Patient selection

We analyzed 69 patient trios of primary CRC and matched metastases and normal tissue using a custom capture-based deep sequencing assay (IMPACT, see Methods). The assay covers all protein-coding exons of 230 actionable or cancer-related genes (mean target coverage 692X). Only microsatellite-stable tumors were included in the study. Sixty-two (90%) patients presented with stage IV disease (Table 1). In 52 (75%) patients, the primary tumor and metastasis were resected at the same time (concurrent). Among the remaining 17 cases the mean interval time between resections was 15.3 months. Thirty (43%) patients were chemonaive prior to resection (Table 1). None of the treated patients received an anti-EGFR therapy prior to resection.

**Table 1:** **Clinical characteristics of CRC cases subjected to targeted sequencing**

**Clinical characteristic**	**N = 69**
Median age, years	59
Range	26-88
Sex	
Male	34 (49%)
Female	35 (51%)
Primary tumor location	
Right	28 (40%)
Left	30 (44%)
Rectum	11 (16%)
Stage at presentation	
I	0 (0%)
II	4 (6%)
III	3 (4%)
IV	62 (90%)
Resection	
Concurrent	52 (75%)
Subsequent	17 (25%)
Treatment	
Chemonaive	30 (43.5%)
Prior treatment	39 (56.5%)
Both tumors	27 (39%)
Primary only	1 (1.5%)
Metastasis only	11 (16%)

### Mutation profiles are highly concordant between primary and metastatic tumors

**Figure 1:**
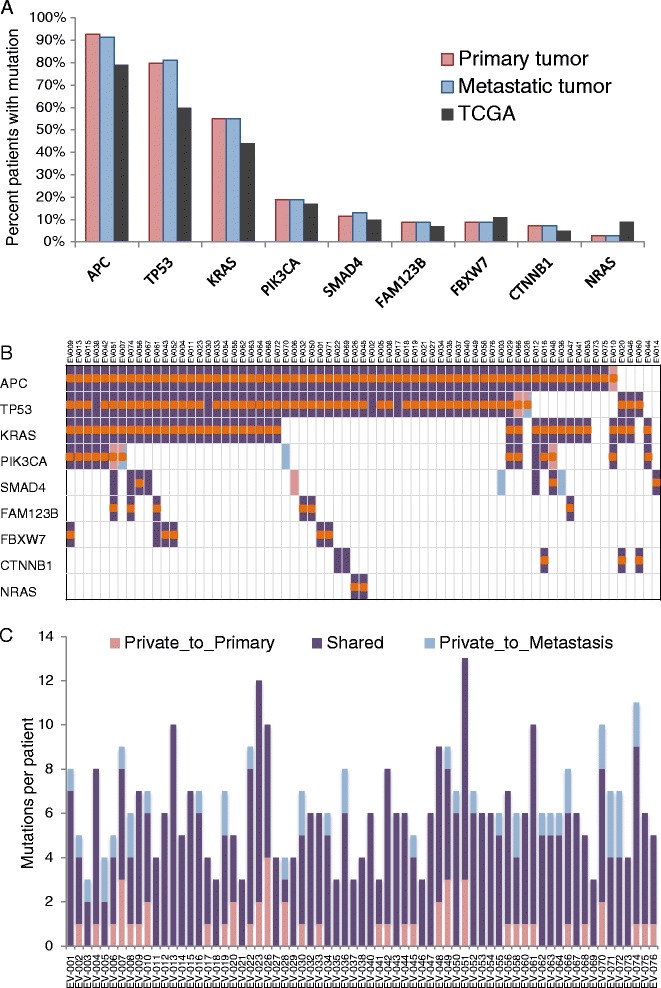
**Mutation patterns are concordant between primary and metastatic tumors and consistent with TCGA. (A)** Most commonly mutated gene frequencies are similar to those of the TCGA non-hypermutated cohort, with minor differences likely due to increased sequencing depth and more advanced disease. **(B, C)** Mutations are highly concordant between primary and metastatic tumors. Shared mutations are in dark purple, private to primary is light red, private to metastasis in light blue. Mutations that are loss-of-function (nonsense, frameshift, or splice site) or that occur in at least five samples in Cosmic are marked with an orange dot.

**Figure 2:**
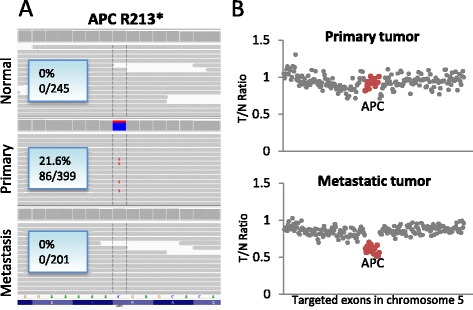
**Phenotypic concordance of mutations.** In patient 10, the primary tumor harbors a nonsense mutation not found in the metastatic tumor **(A)**. **(B)** However, the tumor/normal ratio per exon of coverage on chromosome 5 shows that the exons of APC (red dots) are deleted in the metastatic tumor, yielding identical phenotypic results. Similar results were found for PIK3CA and TP53 (Additional file 2: Figure S2).

Overall, we detected 434 distinct non-synonymous somatic mutations and indels (Additional file 1: Table S1). The mutation profile was consistent with the expected mutation frequencies for non-hypermutated samples reported by The Cancer Genome Atlas (TCGA) [7] (Figure 1A). We observed APC and TP53 alterations at higher prevalence than the TCGA reported, whereas NRAS mutations were observed less frequently in our study compared to the TCGA. Of the 434 total mutations, 344 (79%) were shared between patient-matched tumors (Figure 1B-C, Additional file 2: Figure S1). No discordant mutations were observed in KRAS, NRAS, or BRAF in our cohort. Further, among the mutations in the genes reported by the TCGA to be significantly mutated in non-hypermutated tumors (Figure 1B; 247/434, 57%), there was very high (93%, 229/247) concordance between primary tumors and matched metastases. The 18 private mutations, defined as mutations called only in the primary or the metastatic tumor, were found in APC (n = 7), PIK3CA (n = 5), SMAD4 (n = 3), and TP53 (n = 3). The majority (n = 5) of the private mutations in APC were secondary mutations in cases that shared a clonal APC mutation. One ‘primary-specific’ event was detectable on further review at a low frequency in the metastasis by comparative analysis. In the remaining case, an APC mutation private to the primary tumor was likely lost in a chromosomal deletion in the paired metastasis (Figure 2). Our findings confirm that genetic alterations that occur early in colorectal carcinogenesis, namely mutations in APC, KRAS, NRAS, and BRAF, persist through tumor evolution and show an exceedingly high level of concordance between primary tumor and metastases [8].

Despite the overall high concordance in mutation profiles we observed, we chose to further investigate the remaining discordance. To determine whether any of this discordance could be explained by intra-tumor heterogeneity, we sequenced 97 additional samples from spatially separate regions of the primary tumors (n = 62) and metastases (n = 37) from 22 patients, encompassing 46 discordant mutations. All 97 samples were derived from formalin fixed paraffin embedded (FFPE) tissue and reviewed for morphology and tumor cellularity. Sequencing of multiple regions and samples resolved 17/46 discordant mutations, including 12/22 (55%) mutations that were originally detected only in the metastasis but were subsequently found to be subclonal in the primary tumor (Additional file 1: Table S1). Altogether these results suggest that the small proportion of discordant mutations we observed may itself be an overestimate of the true discordance when accounting for intra-tumor heterogeneity.

### Private oncogenic alterations are occasionally detected

Convergent phenotypic evolution was observed in two patients harboring distinct mutations in individual genes, one involving independent mutations in TP53 (R248Q and Y163*) and the other involving separate hotspot mutations in PIK3CA (E542K and E545K, Additional file 2: Figure S2). Three additional private events were observed in PIK3CA including two E545K mutations specific to the primary tumor and one in-frame deletion at N107 specific to the metastasis. Private mutations of unknown significance were also found in PIK3CD, PIK3CG, PIK3C2G, PIK3R1, and PTEN. Four of these PI3K pathway events were found in other regions, indicative of subclonality. These results suggest that despite the overall high level of genomic concordance between primary and metastatic CRC, heterogeneity in potentially actionable genes, such as those within the PI3K pathway, is present in at least a subset of patients. This finding could have therapeutic implications given the ongoing evaluation of PI3K inhibitors in clinical trials as well as the potential benefit of aspirin therapy in CRC patients harboring PIK3CA mutations [9].

**Figure 3:**
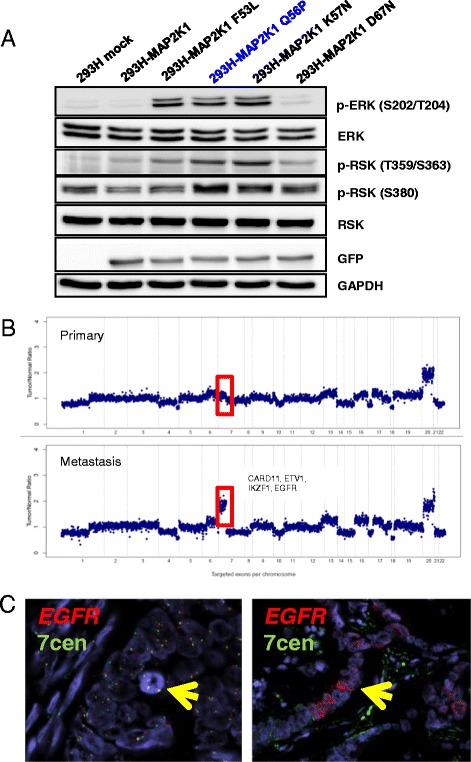
**Metastatic-specific RTK-RAS activating events in RAS/RAF wildtype tumors.** In several tumors lacking KRAS, NRAS, or BRAF mutations, additional events in the RTK-RAS pathway were identified. **(A)** In patient 19, a metastatic-specific MAP2K1 p.Q56P mutation was identified. Transfection of GFP-tagged MAP2K1 plasmids demonstrate that the p.Q56P mutation hyperactivates downstream signaling to the same level as the known p.K57N mutation. **(B)** In patient 3, chromosome 7p is specifically amplified in the metastatic tumor. **(C)** FISH analysis confirms regions of high level amplification of EGFR in the metastatic tumor (right) while the primary tumor only shows 7p polysomy (left).

Notably, we also found genetic events private to the metastasis in three patients lacking KRAS, NRAS, and BRAF mutations. In two cases, MAP2K1 (MEK1) mutations (A106T and Q56P) were detected only in the metastatic sample (Additional file 2: Figure S3). While A106T has not been described, Q56P is a recurrent mutation. Transfection of a GFP-tagged MAP2K1 Q56P plasmid into human embryonic kidney 293H cells resulted in hyperactivation of ERK signaling to a similar degree as other known activating mutations of MAP2K1 (Figure 3A). By contrast, transfection of A106T did not affect signaling (Additional file 2: Figure S4). In another patient, we found a gain of chromosome 7p, which included the CARD11, ETV1, IKZF1, and EGFR genes in the metastasis but not in the primary tumor (Figure 3B). To explore this finding, we performed FISH analysis of both samples. Both the primary tumor and metastasis displayed cells with 7p polysomy; however, the metastasis also contained regions of high-level EGFR amplification not observed within any region of the primary tumor (Figure 3C). These findings collectively suggest that in the absence of RAS mutations, analysis of a metastasis may reveal potentially actionable alterations within other members of the EGFR-RAS signaling pathway.

### Clinical correlates of mutational concordance

We next examined whether mutational concordance was correlated with major clinical characteristics (Additional file 3: Table S2). We observed no significant difference in the number of mutations or degree of mutational concordance for cases with different primary tumor locations (right colon, left colon, or rectum) or for cases with different time intervals between primary and metastasis resections (concurrent *versus* subsequent; Additional file 4: Table S3). We also observed no disease-specific survival differences for patients with concordant and discordant mutation profiles (Additional file 2: Figure S5). However, among patients whose tumors were concurrently resected, those that did not receive prior treatment were more likely to harbor discordant mutations (22/28, 79%) compared to those that received prior therapy (11/24, 46%; chi-square *P* = 0.01. Patients without prior therapy also had a higher total number of mutations, although the difference was not statistically significant (6.6 *vs.* 5.8, *P* = 0.1). These differences may be due to either general tumor debulking and/or decreased tumor heterogeneity from effective drug treatment [10]. In accordance with this hypothesis, the primary tumor size in patients receiving prior treatment was slightly smaller than in chemonaive patients (4.2 *vs.* 5.2 cm, T test *P* = 0.02). Additionally, pre-treated patients with concurrently resected tumors were less likely to harbor primary-only mutations than patients with subsequent resections where only the metastasis received treatment (6/24, 25% *vs.* 7/11, 64%; chi-square *P* = 0.03).

### Whole genome analysis reveals consistent mutation patterns

While our capture-based assay encompasses all well-established targetable or actionable genes in CRC, this approach by definition would fail to identify discordant mutations not included in the assay design. To determine whether our results were representative of the level of mutational concordance genome-wide, we performed whole genome sequencing (WGS) of two cases where the primary tumor and metastasis were concordant by IMPACT analysis (patients 14 and 54) and two cases that harbored discordant mutations (patients 3 and 19, with one and three discordant mutations by IMPACT, respectively). Given the slight disparity in mutations for pre-treated *versus* chemonaive tumors, we chose the patients such that within each group, one patient’s tumors were chemonaive (patients 3 and 54) and the other patient’s tumors had received prior treatment (patients 14 and 19). Primary and metastatic tumors were sequenced to >80× coverage, and matched normal controls were sequenced to >40× coverage. All SNVs and indels detected at higher than 5% allele frequency by IMPACT were detected in our WGS analysis, and no additional mutations in the 230 genes were identified, providing independent validation of the results produced by both methods.

**Figure 4:**
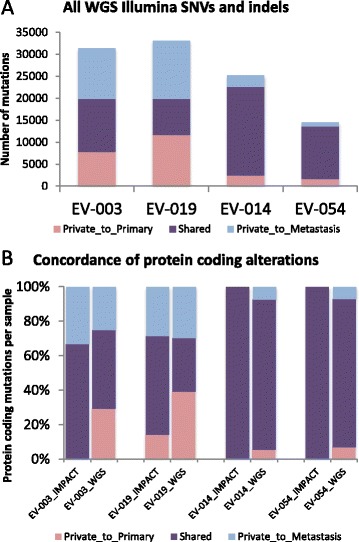
**Whole genome analysis of mutational concordance. (A)** Concordant and discordant non-synonymous mutations and indels for four CRC patients. **(B)** Percent of protein coding alterations per sample for IMPACT and WGS results. Patients 3 and 19 were discordant by IMPACT and remain so by WGS, while patients 14 and 54 remain largely concordant.

The two patients whose primary and metastatic tumors exhibited discordance by IMPACT analysis displayed significant discordance at the WGS level as well (Figure 4, Additional file 2: Figure S6). Comparing all somatic mutations genome-wide, patients 3 and 19 had only 38% and 25% shared mutations, respectively. This rose only slightly to 46% and 32% when considering only non-synonymous exonic mutations (Additional file 5: Table S4). Patient 19 harbored nine loss-of-function mutations (nonsense, frameshift, or splice site) private to the metastasis. Four other metastasis-specific events, including MAP2K1 Q56P detected by IMPACT, are represented in the COSMIC database. Patient 3 harbored six metastasis-specific loss-of-function mutations and three metastasis-specific recurrent mutations also present in COSMIC. A novel PTK7 Q304* mutation was particularly noteworthy, as this protein inhibits canonical Wnt signaling through binding of the frizzled receptor [11]. However, no nonsense mutations were reported in PTK7 in TCGA for CRC, and thus this event, though potentially functional, is rare in CRC. Patient 3 also harbored a metastasis-specific missense mutation in the transcriptional regulation domain of SMAD3 in addition to a metastasis-specific SMAD4 mutation detected by IMPACT.

In contrast, WGS of the paired tumors from the two patients who exhibited 100% concordance by IMPACT analysis (patients 14 and 54) showed a high degree of similarity, with 80% and 83% shared mutations overall. As above, the level of concordance increased even further upon restricting the analysis to non-synonymous protein-coding mutations (87% and 86% shared) and loss-of-function mutations (91% and 91% shared). For patient 54, a single discordant mutation in RUNXT1 was the only discordant alteration present in COSMIC, and no discordant mutations were nonsense or splice-site mutations. Similarly none of the discordant mutations in patient 14 are currently represented in the COSMIC database. Case 14 was notable in that analysis by IMPACT revealed no mutations in both the beta-catenin and RAS pathways, although the tumor harbored an amplification of CDK8, which has be shown to dysregulate the WNT/beta-catenin pathway [12]. No additional beta-catenin pathway alterations were identified by WGS; however, two nonsense mutations in RASA1 (C372* and R679*) were detected in the both primary and metastatic samples. RASA1, a negative regulator of RAS, was altered in 2% of CRC TCGA samples, with mutations in this gene mutually exclusive of KRAS and NRAS mutations. This result prompted the addition of capture probes for the RASA1 gene in our current version of IMPACT and highlights the utility of whole genome sequencing to identify rare alterations in driver pathways such as RAS in tumors pan-negative for the more commonly mutated genes.

## Discussion

We performed deep sequencing of 230 cancer-associated genes in 69 primary CRC tumors and matched metastases to define the mutational concordance of these genes in primary and metastatic tumors. We identified a pattern of genomic alterations consistent with prior studies of CRC, including frequent mutations in the *APC*, *TP53*, and *KRAS* genes [4,7]. Mutations in TP53 were more prevalent in our series than TCGA. TCGA analyzed only primary tumors, the majority of which were derived from patients with stage 1 to 3 disease, whereas all patients in our cohort by definition had metastatic disease. This difference is likely the basis for both the higher prevalence of TP53 mutation and the lower prevalence of NRAS mutations in our cohort *versus* TCGA. The higher prevalence of certain mutations observed from IMPACT may also be the result of greater sensitivity for mutation detection afforded by deeper sequence coverage (mean coverage of 692X). Although we did not observe significant differences in the degree of heterogeneity between those tumors that were resected concurrently or subsequently, future studies are required to determine the extent to which systemic treatments result in preferential selection of specific mutational events.

The only major clinical feature that seemed to correlate with mutational concordance was whether the tumors had received prior treatment. In patients where tumors were concurrently resected, chemonaive tumors were more likely to have private mutations than pre-treated tumors. Similarly, patients with pre-treated metastatic tumors subsequently resected were more likely to harbor primary-only mutations than patients with pre-treated concurrent resections. These findings raise the possibility that prior treatment results in a decrease of apparent tumor heterogeneity, though larger studies are needed to further explore this hypothesis.

Overall, our exon capture data indicate a high degree of concordance in mutational profiles, especially when considering events that occur early in colorectal carcinogenesis. Further, multiple examples of discordance involved parallel evolution demonstrated by independent distinct mutations in the same gene [13]. A subsequent whole genome analysis of four paired primary and metastatic samples further suggested that concordance by IMPACT is a good surrogate of genome-wide mutational concordance. Notably, mutations in KRAS, NRAS, and BRAF were 100% concordant between primary tumors and metastases in our cohort, which is consistent with prior studies [6,14-16]. These results are also consistent with the recommendation that molecular testing of the primary tumor is appropriate in most clinical scenarios. Exceptions would include patients with a history of multiple primary tumors or polyps and patients in which interval drug treatment may result in clonal selection of clinically actionable mutations [6]. An example of the latter would be treatment with anti-EGFR therapy, which has been shown to promote the selection of RAS mutant subclones in tumors that are otherwise KRAS, NRAS, and BRAF wild-type [17,18]. In primary tumors lacking KRAS, NRAS, or BRAF mutations, we did identify occult alterations in the EGFR/RAS pathway that in some patients were private to the metastatic tumor. Discordant mutations were also occasionally observed in components of the PI3K pathway, which could be clinically important as selective inhibitors of the pathway are being actively studied in patients with CRC.

Finally, we found that targeted sequencing was sufficient to identify the most clinically actionable alterations found by WGS. This result suggests that an updated IMPACT test may be a viable time- and cost-saving alternative to WGS for molecular profiling of CRC patients.

## Conclusions

Inter- and intra-tumor heterogeneity, a growing concern in the molecular diagnostics field, is apparent in CRC. However, in patients who have not been treated with anti-EGFR therapies, the current clinically actionable genes, KRAS, NRAS, and BRAF, are 100% concordant between primary and metastatic tissues. As the mutational status of these genes guides current clinical practice, diagnostic testing from either tissue site as available is appropriate in most clinical scenarios. Additionally, targeted sequencing is becoming a more common practice in research and clinical settings, and our results demonstrate the clinical utility of this approach both through comparison of primary and metastatic tissue and similarities of results to whole genome sequencing.

## Methods

### Samples

With Institutional Review Board approval (WA0129-12) and compliance with the Helsinki Declaration, we analyzed 69 matched trios (normal, primary, and metastatic tissues) from patients undergoing resection at our institution where frozen tissue was available. The set was enriched for patients with stage IV disease at diagnosis to decrease potential discordant alterations caused by time. All specimens were reviewed for histological verification of a colorectal adenocarcinoma diagnosis and to ensure greater than 50% tumor content. Macrodissection was performed on specimens with less than 50% viable tumor to minimize stromal contamination. Normal DNA was obtained from normal colon tissue located at least 15 cm away from the tumor. Normal and tumor DNA were extracted from shaved sections cut from frozen tissue blocks using the Qiagen DNeasy Blood & Tissue Kit. In two cases where the frozen section contained an adenoma, invasive regions of the primary were macrodissected from formalin-fixed paraffin-embedded sections, and DNA was extracted using the Qiagen DNeasy Blood & Tissue Kit modified for deparaffinization and these sequencing results were used in place of the original frozen data. Additional regions for determination of private mutations were selected from available FFPE tissue blocks and similarly macrodissected and prepared.

### Microsatellite testing

The microsatellite instability (MSI) status was determined for each case using a 5-microsatellite marker (BAT25, BAT26, D17S250, D2S123, and D5S346) genotyping platform according to a standard protocol [19]. Fluorophore-labeled primers were designed (Applied Biosystems) targeting the five loci. All microsatellite loci were amplified for matched normal and tumor DNA in a multiplex polymerase chain reaction (PCR) and submitted for genotyping to the MSKCC Genomics Core. Microsatellite marker stability was analyzed using Peak Scanner™ software. MSI status was categorized as microsatellite stable if all markers were stable, MSI-low if <30% of markers were unstable, and MSI-high if ≥30% of markers were unstable.

### IMPACT targeted sequencing

Library preparation and sequence analysis is as previously described [20]. Briefly, 100 to 500 ng DNA from frozen or formalin fixed paraffin embedded (FFPE) tissue was prepared using NEBNext DNA Library Prep Kit for Illumina with Kapa HiFi DNA Polymerase for PCR steps. A total of 100 ng resulting library was pooled in sets of 12 to 24 samples for capture with custom Nimblegen probes. Each pool was sequenced in a single lane of an Illumina HiSeq 2000. Resulting fastq files were aligned according to best practice with BWA [21], GATK, Picard [22], and Samtools [23]. Mutations and indels were called with Mutect [24] and GATK SomaticIndelDetector, respectively, and annotated with Oncotator [25] and Cosmic v65 [26]. Only non-synonymous mutations above 5% allele frequency in exonic regions were retained. For primary or metastasis-specific mutations, the allele counts at the corresponding site in the alternate sample were examined. If the mutant allele was present in at least 3 total reads and at least 2% of all reads, the event was labeled as ‘both’ primary and metastasis by comparative analysis. Copy number analysis was performed using average read depth from GATK, loess-normalized for GC content, and compared to diploid normal.

### Cell lines and culture

Human embryonic kidney 293H cells were maintained in DME-HG medium supplemented with 10% FBS, 2 mM glutamine, and 50 units/mL each of penicillin and streptomycin. *MAP2K1* mutations were generated from the MEK1-GFP plasmid (Addgene, 14746) using the QuickChange Site-Directed Mutagenesis Kit (Stratagene) as recommended. All mutant plasmids were verified by DNA sequencing. 293H cells were seeded for 70% to 90% confluency at the time of transfection in the culture medium without penicillin and streptomycin overnight. Cells were transiently transfected with wild-type or mutant *MAP2K1* DNA using the Lipofectamine 2000 Transfection Reagent as recommended.

### Western blot analysis

At 24 h post transfection, cells were collected and lysed in 1% NP-40 lysis buffer and processed for immunoblotting as previously described [27]. Rabbit polyclonal antibodies recognizing phosphorylated Erk1/2 (Thr202/Tyr204), Erk1/2, phosphorylated p90RSK (Thr359/Ser363), and phosphorylated p90RSK (Ser380) were obtained from Cell Signaling. Rabbit monoclonal antibodies recognizing RSK1/2/3, GFP, and GAPDH were obtained from Cell Signaling. After incubation with horseradish peroxidase-conjugated secondary antibodies, proteins were detected by chemiluminescence (SuperSignal West Dura Chemiluminescent Substrate, Thermo Scientific) and visualized using the Fuji LAS-4000 (GE Life Sciences).

### Fluorescence *in situ* hybridization

Fluorescence *in situ* hybridization was performed by the MSKCC Molecular Cytogenetics Core using BAC clone RP11-339 F13 and PAC clone RP5-1091E12 spanning the *EGFR* locus in 7p11, both labeled by nick translation with Red dUTP, together with a chromosome 7 centromeric repeat probe (p7t1) labeled with Green dUTP (Enzo Life Sciences, supplied by Abbott Molecular). Initial hybridization showed weak FISH signals against a high level of background autofluorescence. Fresh probe was prepared and new slides were hybridized. FISH signals for the second hybridization were much stronger but the background autofluorescence was still high. Images stacks (9× 0.5 um slices for Red & Green) were captured through the depth of the tissue for recording and analysis. R/G signal ratios were scored in representative tumor fields with a minimum of 30 nuclei, where possible.

### Whole genome sequencing

Five micrograms of DNA from patient-matched frozen primary tumor, metastatic tumor, and normal tissue for EV-003, EV-014, EV-019, and EV-054 were sent for whole genome sequencing at the New York Genome Center (NYGC) and Illumina. Median coverage was 87X for tumors and 50X for normals. Mutations were called on BAM files produced by the Illumina CASAVA alignment pipeline, using Strelka [28]. These mutations were then genotyped on BAM files produced by the BWA aligner, using GATK Unified Genotyper [21,29]. Calls with fewer than 3 reads or 5% frequency in either the primary and metastatic tumor were removed, as well as those that were less than five times the frequency in the normal tissue. Remaining calls were annotated for the canonical isoform using Oncotator and COSMIC v65 [26].

### Data availability

Data are publically available through dbGaP (accession phs000790.v1.p1) and the Memorial Sloan Kettering Cancer Center cBioPortal for Cancer Genomics ([30]; study ‘Colorectal Adenocarcinoma Triplets’) [31].

## Additional files

Additional file 1: Table S1. Table of all IMPACT mutations in patient trios, with sequencing depth, allele frequency, and whether additional sampling resolved site-specific alterations. Additional file 2: Figure S1. Plotted allele frequencies of IMPACT mutations. **Figure S2.** IGV images of TP53 and PIK3CA convergent alterations. **Figure S3.** IGV image of metastatic-specific MAP2K1 Q56P mutation. **Figure S4.** Western blot analysis of MAP2K1 A106T plasmid. **Figure S5.** Kaplan Meier plot based on mutational concordance. **Figure S6.** IMPACT and WGS summary images for one concordant (patient 54) and one discordant sample (patient 19). Additional file 3: Table S2. Table of patients and associated clinical and molecular data. Additional file 4: Table S3. Tables of number of concordant mutations or samples for site of primary tumor and resection timing. Additional file 5: Table S4. Table of non-synonymous exonic mutations from WGS.

## Abbreviations

CRC: Colorectal cancer
IMPACT: Integrated mutation profiling of actionable cancer targets
MSI: Microsatellite instability
SNV: Single nucleotide variant
TCGA: The Cancer Genome Atlas
WGS: Whole genome sequencing
