# Supplementary material for: Comparative sequencing analysis reveals high genomic concordance between matched primary and metastatic colorectal cancer lesions
Source: Genome Biol. 2014 Aug 28;15(8):454. doi: 10.1186/s13059-014-0454-7 (PMC4189196; doi:10.1186/s13059-014-0454-7)
Supplement: Additional file 2: Figure S1. — Plotted allele frequencies of IMPACT mutations. Figure S2. IGV images of TP53 and PIK3CA convergent alterations. Figure S3. IGV image of metastatic-specific MAP2K1 Q56P mutation. Figure S4. Western blot analysis of MAP2K1 A106T plasmid. Figure S5. Kaplan Meier plot based on mutational concordance. Figure S6. IMPACT and WGS summary images for one concordant (patient 54) and one discordant sample (patient 19). [file 13059_2014_454_MOESM2_ESM.pptx]

## Slide 1
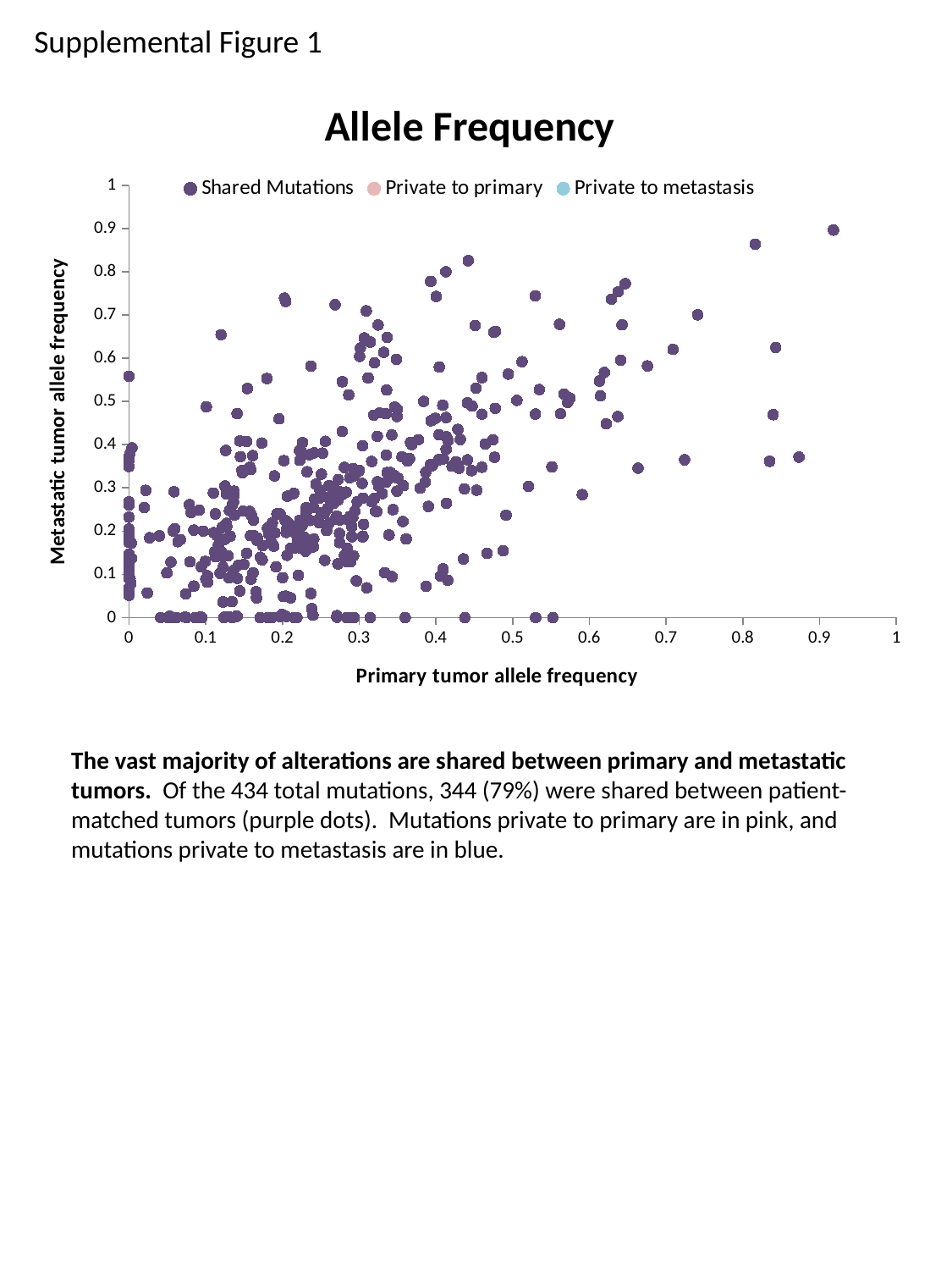

Supplemental Figure 1
### Chart: Allele Frequency
| Category | | Private to primary | |
|---|---|---|---|The vast majority of alterations are shared between primary and metastatic tumors. Of the 434 total mutations, 344 (79%) were shared between patient-matched tumors (purple dots). Mutations private to primary are in pink, and mutations private to metastasis are in blue.

## Slide 2
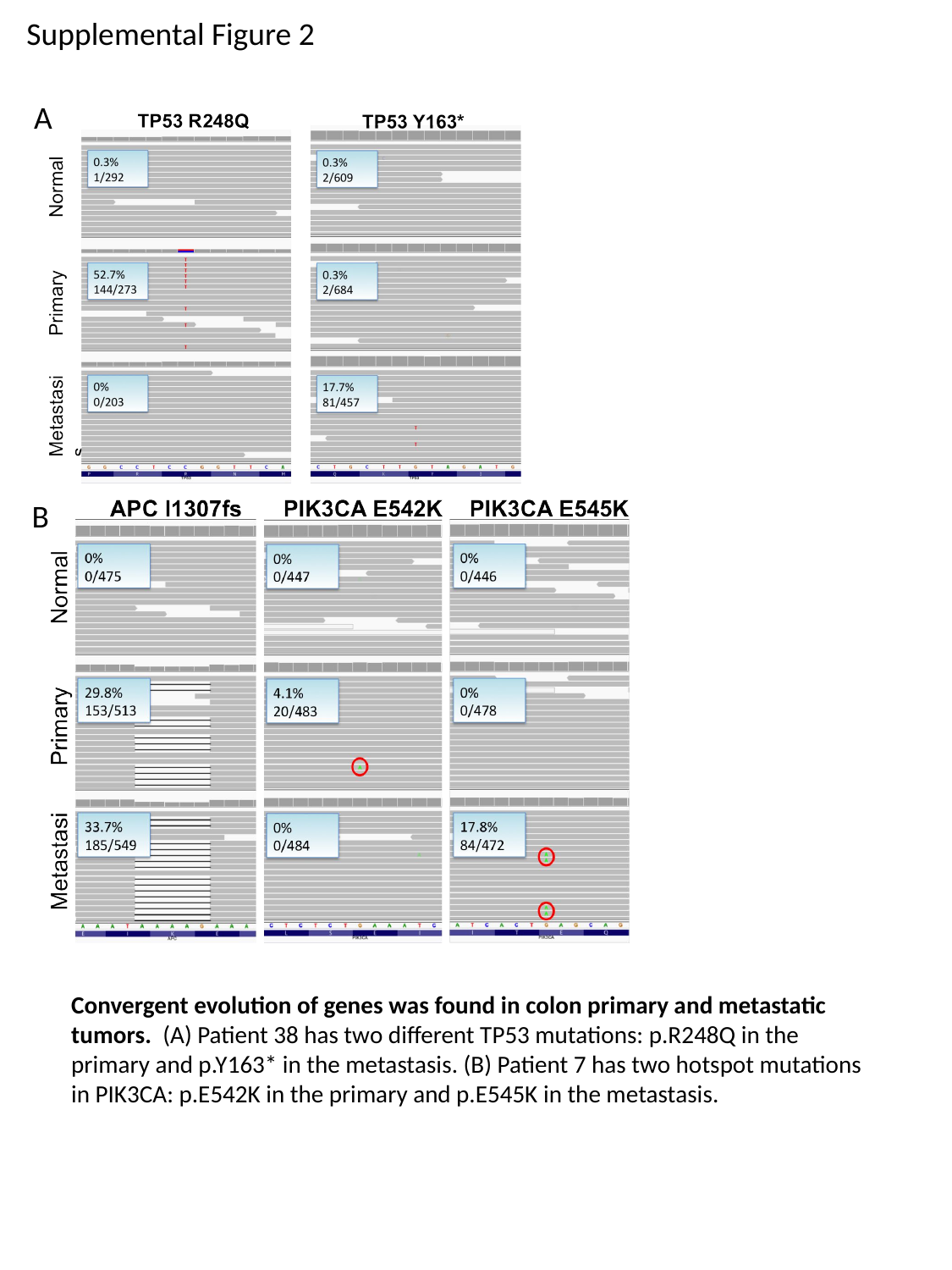

Supplemental Figure 2
A
B
Convergent evolution of genes was found in colon primary and metastatic tumors. (A) Patient 38 has two different TP53 mutations: p.R248Q in the primary and p.Y163* in the metastasis. (B) Patient 7 has two hotspot mutations in PIK3CA: p.E542K in the primary and p.E545K in the metastasis.

## Slide 3
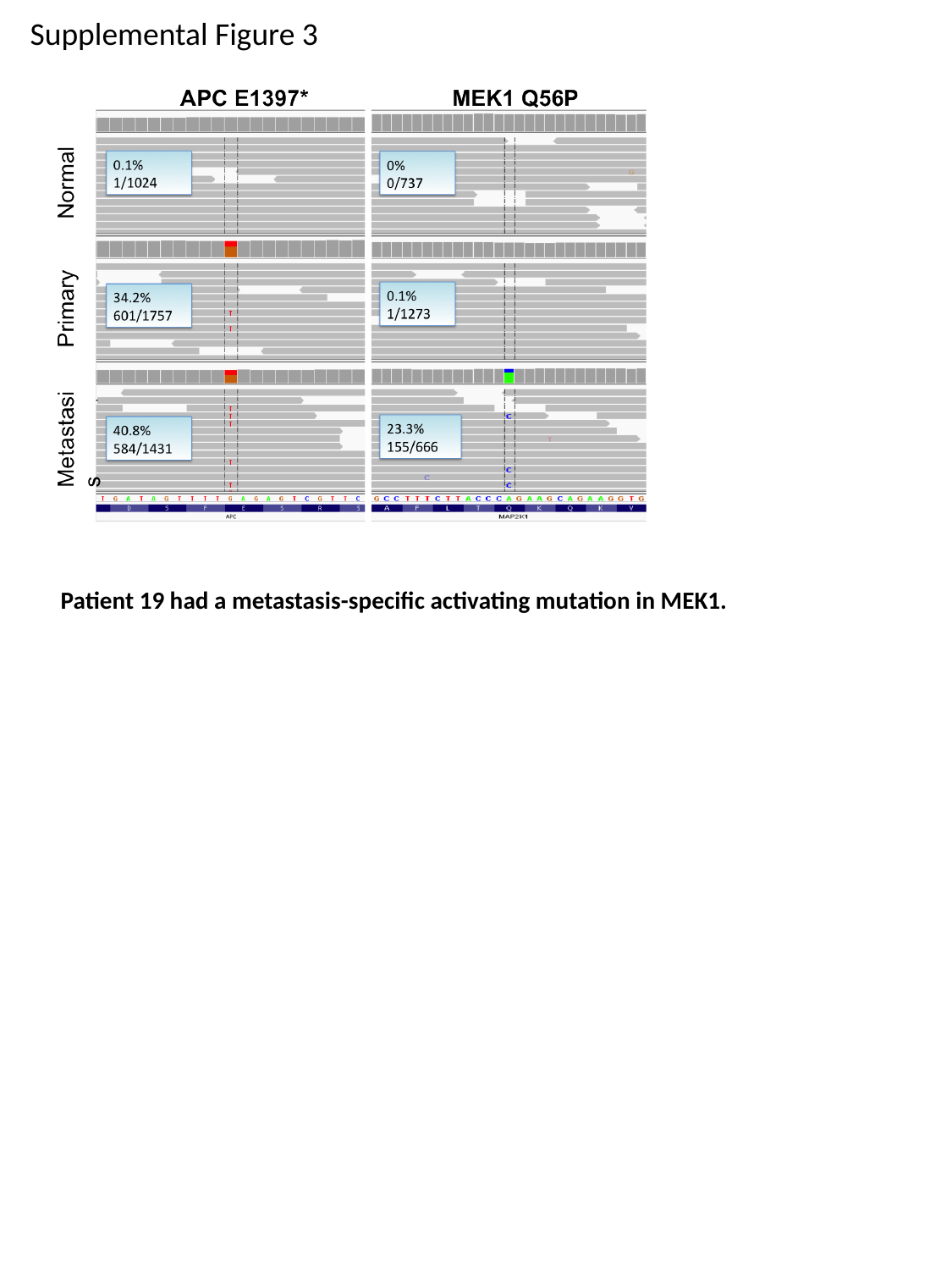

Supplemental Figure 3
Patient 19 had a metastasis-specific activating mutation in MEK1.

## Slide 4
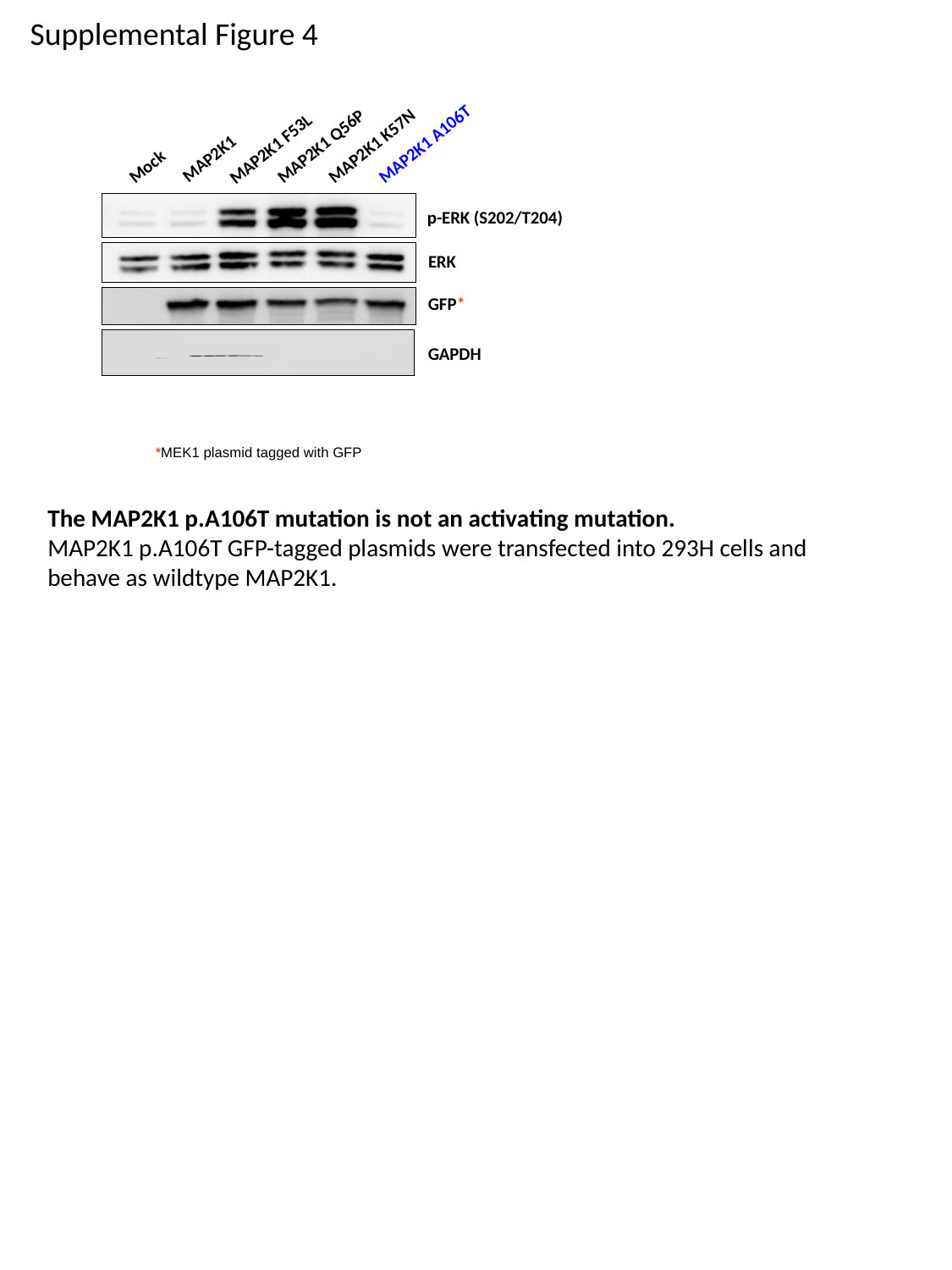

Supplemental Figure 4
MAP2K1 K57N
MAP2K1 Q56P
MAP2K1 A106T
MAP2K1
Mock
MAP2K1 F53L
p-ERK (S202/T204)
ERK
GFP*
GAPDH
*MEK1 plasmid tagged with GFP
The MAP2K1 p.A106T mutation is not an activating mutation.
MAP2K1 p.A106T GFP-tagged plasmids were transfected into 293H cells and behave as wildtype MAP2K1.

## Slide 5
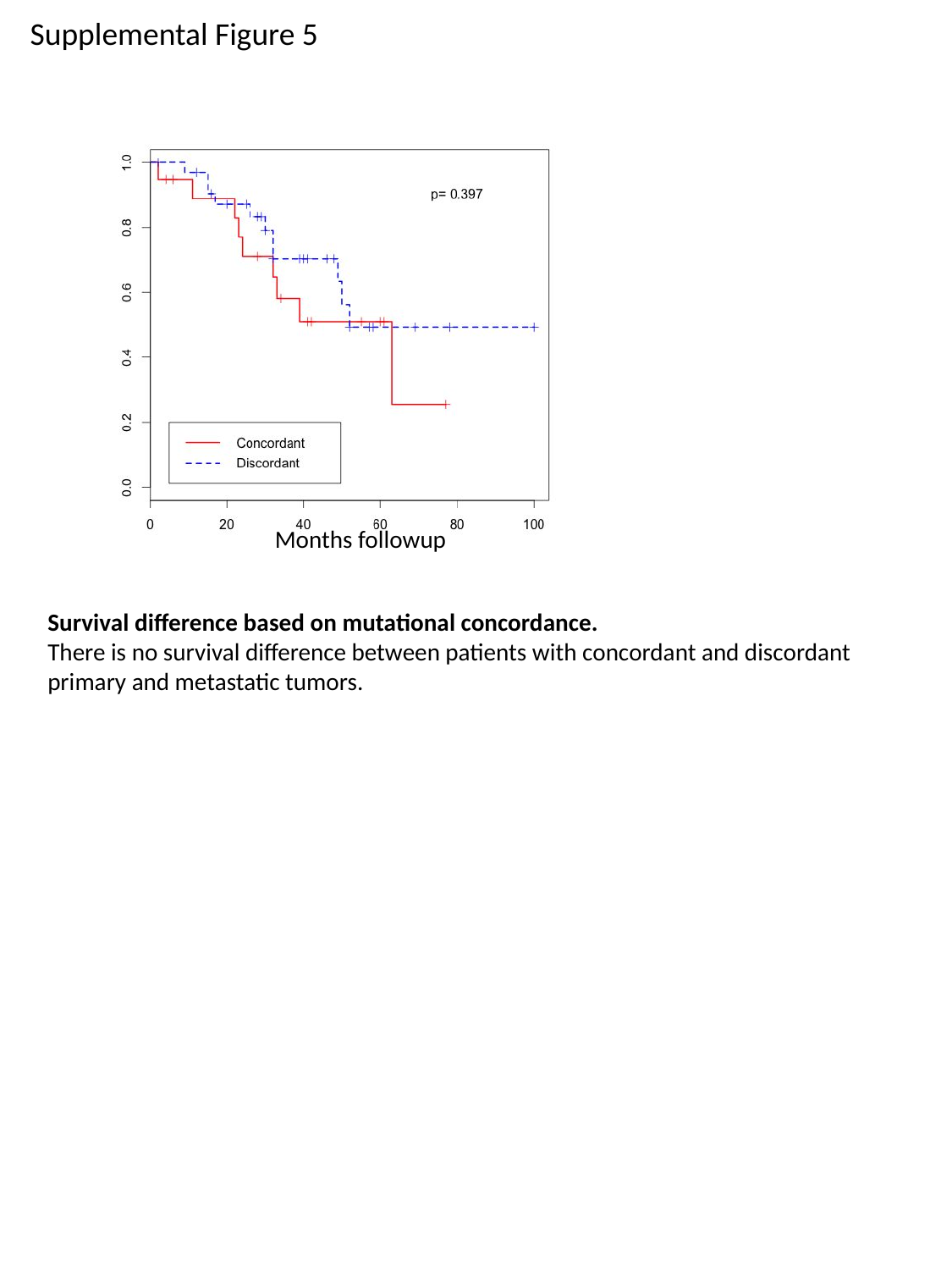

Supplemental Figure 5
Months followup
Survival difference based on mutational concordance.
There is no survival difference between patients with concordant and discordant primary and metastatic tumors.

## Slide 6
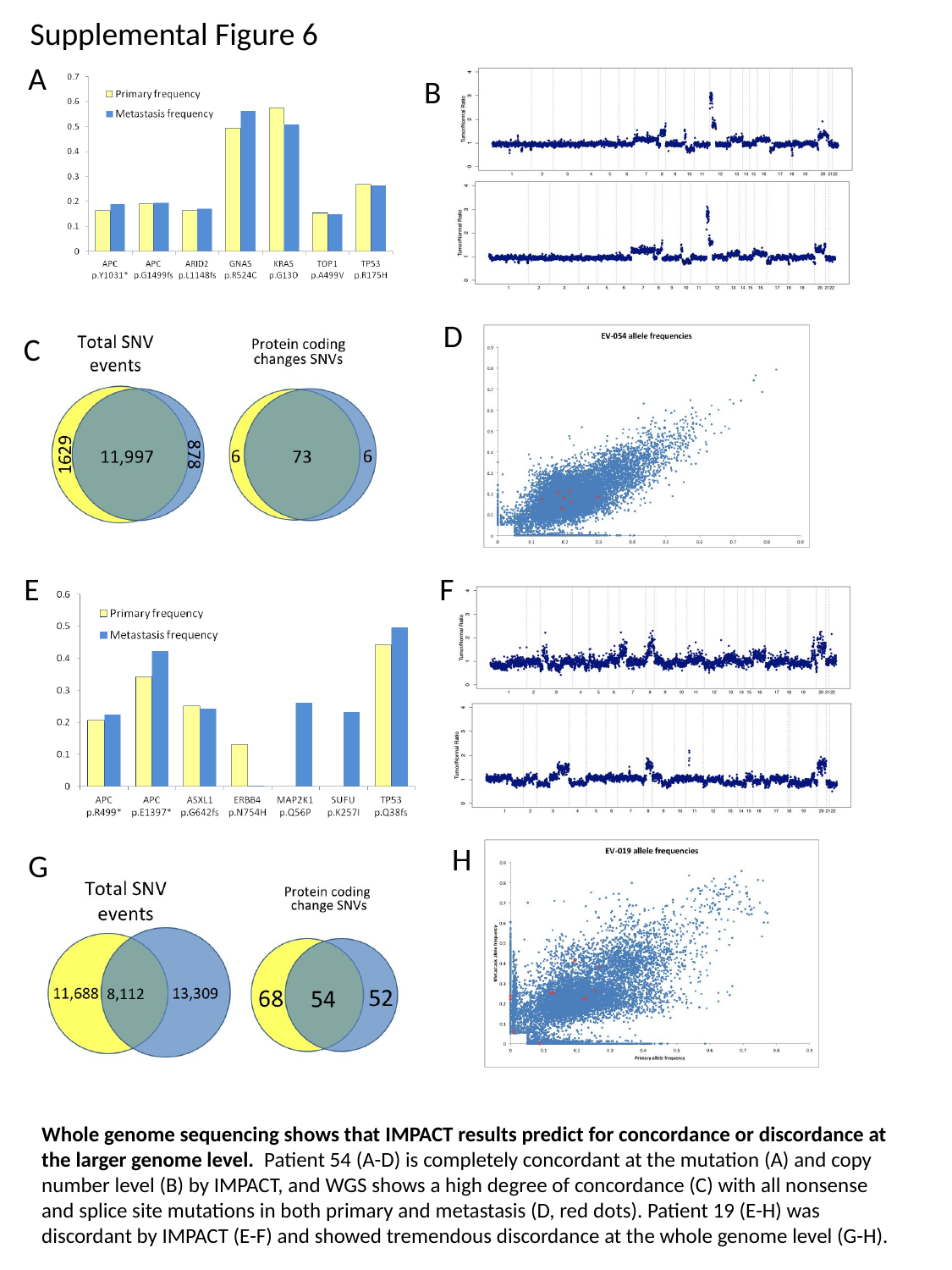

Supplemental Figure 6
A
B
D
C
E
F
H
G
Whole genome sequencing shows that IMPACT results predict for concordance or discordance at the larger genome level. Patient 54 (A-D) is completely concordant at the mutation (A) and copy number level (B) by IMPACT, and WGS shows a high degree of concordance (C) with all nonsense and splice site mutations in both primary and metastasis (D, red dots). Patient 19 (E-H) was discordant by IMPACT (E-F) and showed tremendous discordance at the whole genome level (G-H).
