# Supplementary material for: Comparative sequencing analysis reveals high genomic concordance between matched primary and metastatic colorectal cancer lesions
Source: Genome Biol. 2014 Aug 28;15(8):454. doi: 10.1186/s13059-014-0454-7 (PMC4189196; doi:10.1186/s13059-014-0454-7)
Supplement: Additional file 4: Table S3. — Tables of number of concordant mutations or samples for site of primary tumor and resection timing. [file 13059_2014_454_MOESM4_ESM.docx]

**Supplemental Table 3.** Clinical concordance comparisons.

| Primary tumor location | **Mutations concordant** | **Samples concordant** |
| --- | --- | --- |
| Right | 83%, 161/194 | 37%, 10/27 |
| Left | 76%, 132/174 | 29%, 9/31 |
| Rectum | 77%, 51/66 | 27%, 3/11 |
| Chi-square *P* value | 0.22 | 0.76 |

| Resection timing | **Mutations concordant** | **Samples concordant** |
| --- | --- | --- |
| Concurrent | 80%, 261/327 | 86%, 19/22 |
| Subsequent | 78%, 83/107 | 70%, 33/47 |
| Chi-square *P* value | 0.62 | 0.15 |
